# Anterior chest wall inflammation by whole-body magnetic resonance imaging in patients with spondyloarthritis: lack of association between clinical and imaging findings in a cross-sectional study

**DOI:** 10.1186/ar3551

**Published:** 2012-01-06

**Authors:** Ulrich Weber, Robert GW Lambert, Kaspar Rufibach, Walter P Maksymowych, Juerg Hodler, Anna Zejden, Stefan Duewell, Rudolf O Kissling, Paul L Filipow, Anne G Jurik

**Affiliations:** 1 Department of Medicine, Division of Rheumatology, 562 Heritage Medical Research Building, University of Alberta, Edmonton, Alberta, T6G 2S2, Canada; 2 Department of Rheumatology, Balgrist University Hospital, Forchstrasse 340, CH-8008 Zürich, Switzerland; 3 Department of Radiology and Diagnostic Imaging, University of Alberta, 2A2.41 Walter C Mackenzie Health Sciences Centre, 8440-112 Street, Edmonton, AB, T6G 2B7, Canada; 4 Division of Biostatistics, Institute of Social and Preventive Medicine, University of Zürich, Hirschengraben 84, CH-8001 Zürich, Switzerland; 5 Department of Radiology, University Hospital Zürich, Raemistrasse 100, CH-8091 Zürich, Switzerland; 6 Department of Radiology, Aarhus University Hospital, Noerrebrogade 44, DK-8000 Aarhus C, Denmark; 7 Department of Radiology, Kantonsspital Frauenfeld, Spital Thurgau AG, Pfaffenholzstrasse 4, CH-8500 Frauenfeld, Switzerland

## Abstract

**Introduction:**

Inflammatory involvement of the anterior chest wall (ACW) affects the quality of life of patients with spondyloarthritis (SpA), although involvement of the ACW is often neglected on clinical and imaging evaluation. Whole-body (WB) MRI is an imaging method used to assess the ACW in addition to the sacroiliac joints and spine without inconvenience for patients. Our goals in this study were to describe the distribution of ACW inflammation by WB MRI in both early and established SpA and associations between clinical and imaging findings indicative of inflammation.

**Methods:**

The ACWs of 122 consecutive SpA patients (95 with ankylosing spondylitis (AS) and 27 with nonradiographic SpA (nrSpA)) and 75 healthy controls were scanned by sagittal and coronal WB MRI. The MRI scans were scored independently in random order by seven readers blinded to patient identifiers. Active and structural inflammatory lesions of the ACW were recorded on a web-based data entry form. ACW pain by patient self-report, ACW tenderness on physical examination according to the Maastricht Ankylosing Spondylitis Enthesitis Score (MASES) and lesions detected by MRI were analyzed descriptively. κ statistics served to assess the agreement between clinical and imaging findings.

**Results:**

ACW pain or tenderness was present in 26% of patients, with little difference between AS and nrSpA patients. Bone marrow edema (BME), erosion and fat infiltration were recorded in 44.3%, 34.4% and 27.0% of SpA patients and in 9.3%, 12.0% and 5.3% of controls, respectively. Lesions found by MRI occurred more frequently in AS patients (BME, erosion and fat infiltration in 49.5%, 36.8% and 33.7%, respectively) than in nrSpA patients (25.9%, 25.9% and 3.7%, respectively). The joint most frequently affected by lesions found on MRI scans was the manubriosternal joint. The κ values between clinical assessments and MRI inflammation ranged from -0.10 to only 0.33 for both AS and nrSpA patients.

**Conclusions:**

Among SpA patients, 26% had clinical involvement of the ACW. WB MRI signs of ACW inflammation were found in a substantial proportion of patients with AS (49.5%) and nrSpA (25.9%). There was no association between clinical assessments of ACW, including the MASES, and MRI features.

## Introduction

Inflammatory involvement of the anterior chest wall (ACW) has substantial impact on the quality of life of patients with spondyloarthritis (SpA) [1]. However, ACW involvement is often neglected in clinical assessment. Researchers in uncontrolled studies of ACW inflammation who assessed the association between clinical features and various abnormalities using different imaging modalities have shown inconsistent results regarding the frequency of ACW involvement and associations with imaging findings [2-7]. This may partly reflect the lack of a systematic methodology and inclusion of patients at various stages of disease.

**Figure 1:**
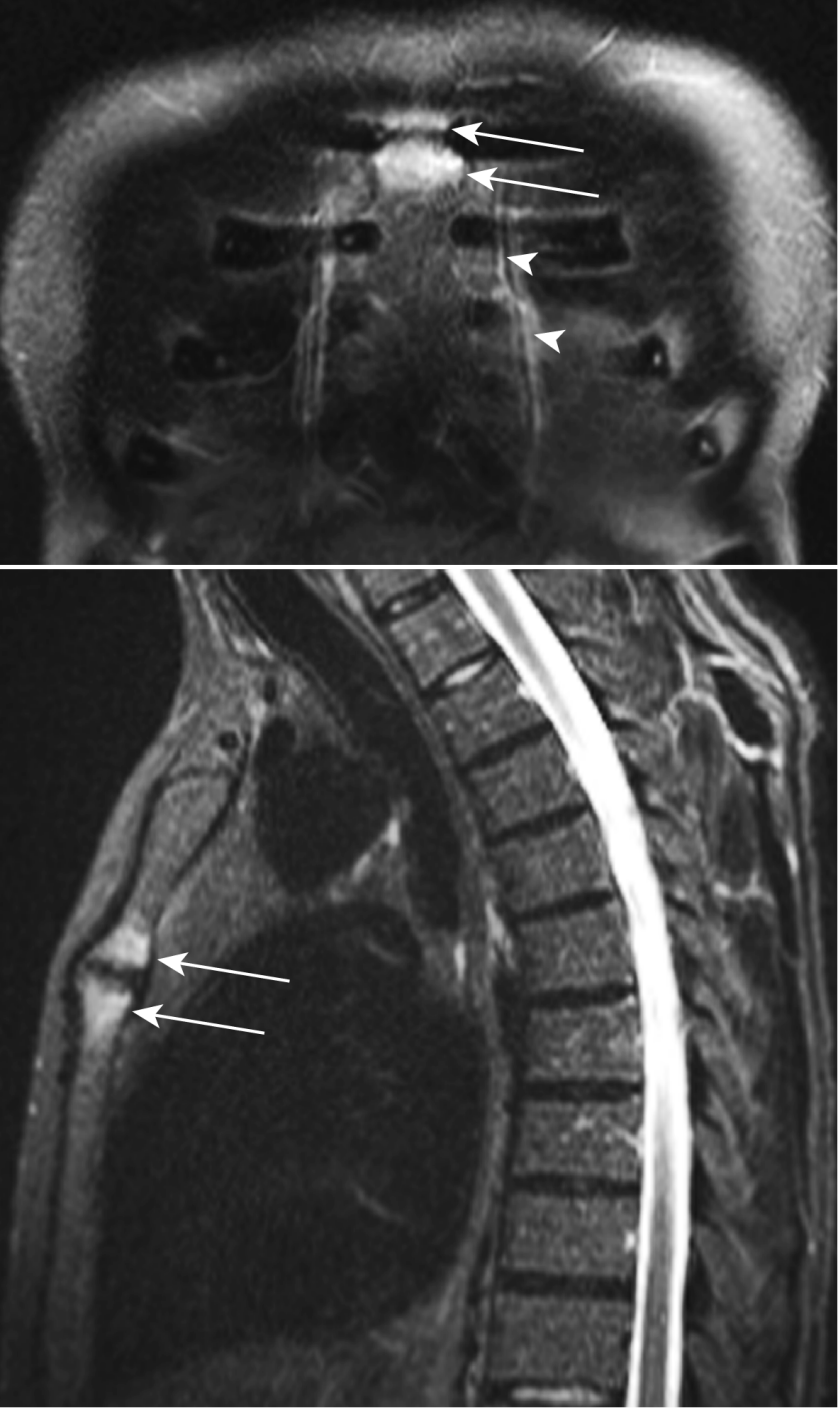
**Inflammatory lesions of the anterior chest wall displayed by whole-body MRI in an AS patient complaining about chest wall pain**. Bone marrow edema (arrows) in both parts of the manubriosternal joint on coronal and sagittal short τ inversion recovery (STIR) sequences in a 46-year-old human leukocyte antigen B27 (HLA-B27)-positive male patient with ankylosing spondylitis and anterior chest wall pain. He had a 6-year history of inflammatory back pain. The arrowheads point to the mammary veins.

**Figure 2:**
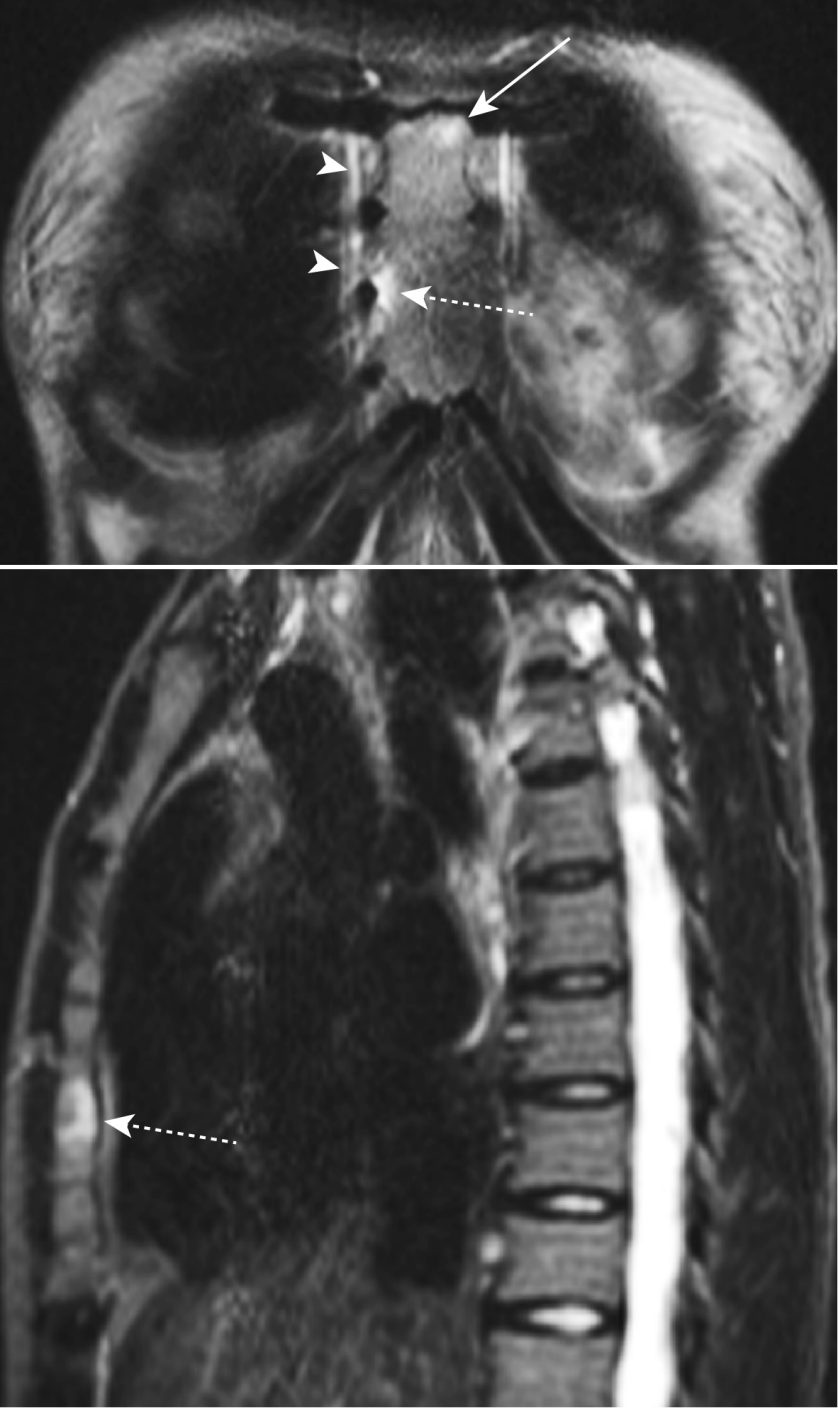
**MRI inflammation of the anterior chest wall in an AS patient without clinical signs of chest wall involvement**. Bone marrow edema (dashed arrows) of the fourth and fifth right sternocostal joints on frontal and sagittal STIR sequences in a 26-year-old HLA-B27-positive female patient with ankylosing spondylitis but no anterior chest wall pain. The patient reported a 2-year history of inflammatory back pain. Bone marrow edema is also seen in the lower portion of the manubriosternal joint on the left side (arrow). The mammary veins are indicated by arrowheads.

Conventional MRI limited to the ACW region is rarely performed in routine clinical practice, even in the presence of ACW pain in SpA patients. Whole-body (WB) MRI is a recently introduced imaging modality based on multichannel technology, which offers an opportunity to evaluate inflammation of the ACW (Figures 1 and 2) in addition to the entire spine, the sacroiliac joints (SIJs) and the shoulder and hip girdle in a single examination that takes about 30 minutes [8-10]. Advantages of MRI over radiography, computed tomography and scintigraphy for imaging the ACW are the concomitant assessment of active and structural lesions, the precise anatomical visualization of inflammatory lesions due to superior spatial and contrast resolution, multiplanar capability and the absence of radiation [11].

The objectives of this study were (1) to conduct a systematic and controlled evaluation of the frequency and anatomical distribution of active and structural lesions detected by MRI in the ACW of patients with ankylosing spondylitis (AS) and nonradiographic axial SpA (nrSpA) and (2) to assess the association between clinical and imaging findings of ACW inflammation.

## Materials and methods

### Patients

In this cross-sectional study, we enrolled 95 patients who met the modified New York classification criteria for AS [12] and 27 patients with nrSpA who fulfilled the Assessment of SpondyloArthritis International Society (ASAS) classification criteria for axial SpA [13] and also had inflammatory back pain according to the Calin criteria [14]. The patients were consecutively recruited from a university rheumatology outpatient clinic (Balgrist University Hospital, Zürich, Switzerland). There were no limitations on the age and disease duration in our patient group representative of patients seen in routine practice in a tertiary care center. Patients undergoing ongoing therapy with and those who had been treated previously with biological agents were excluded from the study. A control group of 75 healthy volunteers was recruited from the staff of the same university hospital that enrolled the SpA patients. The control subjects were selected on the basis of the absence of clinically relevant back pain according to the Nordic questionnaire [15] and had no symptoms of SpA in their medical history. All study subjects underwent a WB MRI examination, including imaging of the ACW in the coronal and sagittal planes. Approval by the local Ethics Committee was obtained (Kantonale Ethikkommission Zürich and Spezialisierte Unterkommission Orthopädie/Bewegungsapparat, EK 37/2004), and all study subjects gave their written informed consent.

### Clinical assessment of anterior chest wall pain

#### Physician assessment

ACW tenderness according to the Maastricht Ankylosing Spondylitis Enthesitis Score (MASES) [16] was assessed in the 122 SpA patients. MASES is the only one of six available enthesitis indices to include tender points of the ACW [17]. Tenderness of the first and seventh sternocostal joints on both sides was recorded dichotomously as present or absent in a diagrammatic ACW scheme. We standardized pressure intensity as that level which elicited pallor of the distal nail bed upon palpation with the patient in a supine position during the examination. A positive physician assessment was defined as tenderness in at least one of the four MASES locations. The physician was not aware of the patient's self-assessment form at the time of the clinical examination or of the WB MRI findings, both of which were performed after the physician assessment.

#### Patient self-assessment

Patients recorded pain in the anterior rib cage on a form representing a manikin. This form was filled in after the clinical consultation if the WB MRI took place the same day or the patient returned the form on the day the WB MRI was scheduled. Patient self-assessment forms were available for only 73 of the 122 SpA patients because the manikin form was introduced two years after start of the WB MRI program. A positive patient assessment was defined as any indication of pain on the manikin form in an area bordered cranially by the sternoclavicular joints (SClJs) and clavicles, caudally by the inferior rib cage and laterally by the anterior axillary line. This extended area for pain records was chosen because referred pain to areas distant from inflamed sternal joints has been well-described [18-20].

### MRI protocol

WB MRI scans are composed of images of the upper and the lower half of the trunk that are fused by dedicated software. To blind the readers regarding inflammation of the lower half of the spine and the SIJ, only the nonfused sagittal and coronal MRI scans of the upper half of the trunk were used for this study. The WB MRI studies were acquired using a MAGNETOM Avanto 1.5 T MRI scanner (Siemens Medical Solutions, Erlangen, Germany). The detailed WB MRI protocol has been described previously [9,10]. Briefly, the following parameters were used to obtain the sagittal images. Turbo short τ inversion recovery (STIR) repetition time (TR) was 6,270 ms, echo time (TE) 93 ms, inversion time (TI) 130 ms, T1-weighted spin-echo (T1SE) MRI sequences TR 401 ms and TE 11 ms. Two sequences were used with a field of view (FOV) of 450 mm and an imaging matrix of 780 × 448 mm (STIR) and 890 × 448 mm (T1SE), 3-mm section thickness and interslice gap 0.3 mm. For coronal sequences the parameters were turbo STIR TR 9,860 ms, TE 99 ms, TI 130 ms. For T1SE, the parameters were TR 571 ms and TE 12 ms. The FOV was 450 × 450 mm for each sequence, imaging matrix 660 × 384 pixels, slice thickness 5 mm and interslice gap 1.0 mm.

### Analysis of MR images (Figures 1 and 2)

STIR and T1SE sequences of sagittal and coronal MRI scans of the ACW were scored independently by seven readers from four institutions (five radiologists: AGJ, AZ, JH, RGL and SD; and two rheumatologists: UW and WPM) blinded to subject identifiers. The films were evaluated in random order on electronic work stations at the institution of each reader.

#### Definitions of lesions of the anterior chest wall detected by MRI

We developed standardized definitions of active and structural inflammatory lesions in the ACW detected by MRI. These focused on bone marrow edema (BME) seen on STIR images; joint erosion and marrow fat infiltration on T1SE images of the SClJ, manubriosternal joint (MSJ) and sternocostal joint (SCoJ); synovitis of the SClJ on STIR sequences; and fusion of the MSJ on T1SE sequences. The primary reference for normal bone marrow signal intensity was the center of the sternal manubrium or body on the STIR sequence. In the setting of diffuse sternal inflammation, the center of a normal thoracic vertebra at the same craniocaudal level constituted the reference normal signal.

BME was defined as bright subchondral bone marrow signal on STIR images. Erosion was defined as a full-thickness defect of articular cortical bone combined with an altered bone marrow signal on T1SE images. Fat infiltration was defined as focal bright subcortical bone marrow signal on T1SE images. Synovitis of the SClJ was recorded when fluid accumulation was present in the joint cavity or extended at least 5 mm lateral to the SClJ. Fusion of the MSJ was defined by bone marrow signal on T1SE images extending partially or completely across the joint space.

#### Reference magnetic resonance image set of the anterior chest wall

A reference MR image set based on the definitions for these five types of acute and structural inflammatory lesions of the three joints of the ACW was developed by consensus among the seven readers. The reference image set also included normal ACW findings and anatomical variants and served to calibrate the observers at the time of reading the study MRI scans.

#### Standardized assessment of lesions of the anterior chest wall detected by MRI and online data entry module

The MRI findings were entered into a customized web-based module displaying a schematic of the three joints of the ACW, which comprised the following eight locations: the clavicular and sternal portion of both SClJs, the upper and lower parts of the MSJ and the right and left SCoJs. BME, erosion and fat infiltration were recorded dichotomously as being present or absent in all eight locations. Synovitis and bone fusion were recorded only for the SClJs and the MSJ, respectively.

### Statistical analysis

#### Frequency of lesions found by MRI

The distribution of the five lesions detected by MRI in patients and controls for the three joints and the entire ACW (at least one of three joints affected) was analyzed descriptively by defining "presence" as an observation recorded concordantly by the majority (at least four of seven) of raters. This approach of reporting the lesion frequency according to the majority of the seven observers was regarded as an optimal balance between sensitivity and specificity to avoid overinterpretation of scattered lesions found by MRI as indicated by a single observer or a minority of readers only.

#### Reliability of lesion detection by MRI

Interobserver agreement was analyzed by the median of the pairwise estimated Cohen's κ values [21] for the 21 possible reader pairs, for all five lesion types and for the three ACW joints, respectively. The interreader agreement was defined as slight, fair, moderate, substantial and almost perfect by values of the estimated Cohen's κ < 0.2, 0.2 ≤ κ < 0.4, 0.4 ≤ κ < 0.6, 0.6 ≤ κ < 0.8 and 0.8 ≤ κ < 1, respectively [22].

#### Agreement between patient and physician assessment, and association between clinical and MRI findings of the anterior chest wall

For those 73 SpA patients for whom both clinical assessments were available, agreement between patient and physician assessments was evaluated by computing Cohen's κ value [21]. The agreement between clinical findings (for both patient and for physician assessment) and lesions of the ACW detected by MRI was analyzed by providing the median of Cohen's κ of all seven individual readers. A positive patient assessment (*n *= 73) or physician assessment (*n *= 122) has been defined above (clinical assessment of ACW pain). Lesions found on MRI (BME, erosion or fat infiltration) were regarded as present if the abnormality was recorded in at least one of eight ACW joint locations by each of the seven readers. This approach to select only lesions observed on MRI studies by all seven readers aimed at high specificity for comparison with the two clinical assessment methods where ACW pain was either present or absent. The agreement was calculated for the SpA, AS and nrSpA groups and for BME, erosion and fat infiltration of the ACW, respectively. The same definitions to interpret the κ values regarding the degree of agreement were applied as described above. It is possible for the κ value to be negative, indicating that there is less agreement than would be expected by chance between two assessment methods.

## Results

### Characteristics of the patient and control group

Demographic and clinical data of the patients and controls are shown in Table 1. The median age of the AS group was 35.7 years, of the nrSpA patients 27 years and of the controls 30.3 years, respectively. 87.1% of the AS patients and 88.9% of the nrSpA patients were human leukocyte antigen B27-positive. The median symptom duration in the AS group was 11 years compared with 1.2 years in the nrSpA group.

**Table 1:** Characteristics of the study subjects^a^

Variable	SpA group (*n *= 122)	AS group (*n *= 95)	nrSpA group (*n *= 27)	Control group (*n *= 75)
Male:female ratio (% male)	90:32 (73.8)	73:22 (76.8)	17:10 (63.0)	42:33 (56.0)
Age, years	33.8 [17.3 to 71.6]	35.7 [17.3 to 71.6]	27.0 [18.8 to 44.8]	30.3 [17.7 to 63.8]
Symptom duration, years	7.0 [0.3 to 41.0]	11.0 [0.3 to 41.0]	1.2 [0.3 to 5.0]	N/A
HLA-B27-positive, *n *(%)^b^	105/120 (87.5)	81/93 (87.1)	24/27 (88.9)	N/A
BASDAI, NRS range 0 to 10	4.4 [0.4 to 9.0]	4.6 [0.4 to 9.0]	3.9 [0.8 to 6.3]	N/A
BASFI, NRS range 0 to 10	2.9 [0 to 8.2]	3.2 [0 to 7.8]	2.1 [0 to 8.2]	N/A
BASMI, NRS range 0 to 10	1.0 [0 to 8.0]	1.0 [0 to 8.0]	0 [0 to 4.0]	N/A
CRP level, mg/L	5.0 [0 to 150.0]	5.0 [0 to 57.0]	3.0 [0 to 150.0]	N/A
ESR (mm/hour)	12.5 [1.0 to 72.0]	14.0 [2.0 to 69.0]	8.5 [1.0 to 72.0]	N/A

^a^AS = ankylosing spondylitis; BASDAI = Bath Ankylosing Spondylitis Disease Activity Index [23]; BASFI = Bath Ankylosing Spondylitis Functional Index [24]; BASMI = Bath Ankylosing Spondylitis Metrology Index [25]; CRP = C-reactive protein, reference range ≤5 mg/L; ESR = erythrocyte sedimentation rate; HLA-B27 = human leukocyte antigen B27; N/A = not applicable; NRS = numeric rating scale; nrSpA = nonradiographic axial spondyloarthritis; SpA = spondyloarthritis. ^b^HLA-B27 was not tested in two patients in the AS group. Values for the continuous demographic and clinical variables are medians [IQR] unless otherwise stated.

### Clinical evaluation of anterior chest wall inflammation by patient and physician assessments

The frequency of ACW pain by patient assessment and of ACW tenderness by physician assessment is displayed in Table 2. In SpA patients, both clinical assessments showed the same frequency of ACW involvement of 26% with little difference between the AS and the nrSpA group despite the different median disease durations of 11.0 versus 1.2 years. The agreement between patient and physician assessments was moderate with an estimated Cohen's κ value of 0.50 (95% CI = 0.24 to 0.71).

**Table 2:** Clinical anterior chest wall inflammation by patient and physician assessment and concordance between the two assessments^a^

Assessments	SpA group	AS group	nrSpA group
Patient assessment, *N*	73	58	15
ACW pain present, *n *(%)	19 (26.0%)	16 (27.6%)	3 (20.0%)
Physician assessment, *N*	122	95	27
ACW tenderness present, *n *(%)	32 (26.2%)	25 (26.3%)	7 (25.9%)
Patient versus physician assessment^b ^(SpA group)	ACW tenderness present	ACW tenderness absent	Total
ACW pain present, *n *(%)	12 (16.4%)	7 (9.6%)	19 (26.0%)
ACW pain absent, *n *(%)	7 (9.6%)	47 (64.4%)	54 (74.0%)
Total, *n *(%)	19 (26.0%)	54 (74.0%)	73 (100%)

^a^ACW = anterior chest wall; AS = ankylosing spondylitis; nrSpA = nonradiographic spondyloarthritis; SpA = spondyloarthritis. ^b^Estimated Cohen's κ = 0.50 (95% CI = 0.24 to 0.71).

### Distribution of inflammatory anterior chest wall lesions found by whole-body MRI

The frequency of inflammatory lesions of the entire ACW in SpA patients scanned by MRI was 44.3% for BME, 34.4% for erosions and 27.0% for fat infiltration, respectively (Table 3). Any of these types of lesions detected by MRI was observed more frequently than the clinical involvement of the ACW in 26% of the SpA patients. All lesion types were recorded more frequently in AS patients than in nrSpA patients: BME in 49.5% versus 25.9%, erosion in 36.8% versus 25.9% and fat infiltration in 33.7% versus 3.7%, respectively. On a joint level, the most frequently affected joint by all three lesions was the MSJ (33.6% for BME, 30.3% for erosion and 20.5% for fat infiltration in the SpA group), followed by the SClJ (23.8%, 11.5% and 10.7%, respectively), whereas the SCoJs were only rarely involved (6.6%, 0% and 3.3%, respectively). Postinflammatory fusion of the MSJ was detected in AS patients only (14.7%). Both sides of the SClJ were equally affected by synovitis in the SpA group (in six and in five patients on the right and the left sides, respectively, and in five patients on both sides).

**Table 3:** Frequency of MRI-detected lesions recorded concordantly by the majority of readers (at least four of seven) for each joint and per subject^a^

Variables	SpA group (*N *= 122)	AS group (*N *= 95)	nrSpA group (*N *= 27)	Control group (*N *= 75)
SClJ bone marrow edema	29 (23.8%)	26 (27.4%)	3 (11.1%)	1 (1.3%)
SClJ erosion	14 (11.5%)	13 (13.7%)	1 (3.7%)	2 (2.7%)
SClJ fat infiltration	13 (10.7%)	13 (13.7%)	0 (0%)	1 (1.3%)
SClJ synovitis	16 (13.1%)	13 (13.7%)	3 (11.1%)	1 (1.3%)
MSJ bone marrow edema	41 (33.6%)	35 (36.8%)	6 (22.2%)	6 (8.0%)
MSJ erosion	37 (30.3%)	31 (32.6%)	6 (22.2%)	7 (9.3%)
MSJ fat infiltration	25 (20.5%)	24 (25.3%)	1 (3.7%)	3 (4.0%)
MSJ fusion	14 (11.5%)	14 (14.7%)	0 (0%)	0 (0%)
SCoJ bone marrow edema	8 (6.6%)	8 (8.4%)	0 (0%)	0 (0%)
SCoJ erosion	0 (0%)	0 (0%)	0 (0%)	0 (0%)
SCoJ fat infiltration	4 (3.3%)	4 (4.2%)	0 (0%)	0 (0%)
ACW bone marrow edema (≥1 joint)	54 (44.3%)	47 (49.5%)	7 (25.9%)	7 (9.3%)
ACW erosion (≥1 joint)	42 (34.4%)	35 (36.8%)	7 (25.9%)	9 (12.0%)
ACW fat infiltration (≥1 joint)	33 (27.0%)	32 (33.7%)	1 (3.7%)	4 (5.3%)
ACW ≥1 of 5 lesions^b ^(≥1 joint)	72 (59.0%)	62 (65.3%)	10 (37.0%)	15 (20.0%)

^a^ACW = anterior chest wall; AS = ankylosing spondylitis; MSJ = manubriosternal joint; nrSpA = nonradiographic axial spondyloarthritis; SClJ = sternoclavicular joint; SCoJ = sternocostal joint; SpA = spondyloarthritis. ^b^Bone marrow edema ACW and/or erosion ACW, fat infiltration ACW, synovitis SClJ, fusion MSJ. The number of patients with MRI-detected lesions in the entire ACW may be lower than the addition of patients with MRI changes of the three joints SClJ, MSJ and SCoJ, because some patients showed inflammation in more than one joint. Values are the number of subjects (percentage).

The seven readers were in substantial agreement for BME of the MSJ, with a median κ of 0.65. Erosions and fat infiltration of the MSJ also showed the highest reliability (median κ 0.46 and 0.47, respectively) compared with the other two ACW joints. The only lesion type showing moderate agreement in the SClJ was BME (κ = 0.49). The poor reliability of all lesion types in the SCoJ computed by κ statistics resulted from the low number of scattered lesions recorded by the seven readers, as κ values depend on the prevalence of the finding under observation [26,27]. The κ values for the most experienced of the 21 possible reader pairs and for the MSJ were 0.78 for BME, 0.73 for erosion and 0.67 for fat infiltration, respectively, indicating substantial agreement for all three lesions.

In the control group, lesions seen on MRI that were compatible with BME were observed in 9.3%, erosions in 12.0% and fat infiltration in 5.3%, respectively. Nearly all lesions reported in healthy controls were seen in the MSJ (8.0% for BME, 9.3% for erosion and 4.0% for fat infiltration), with few lesions in the SClJ (1.3%, 2.7% and 1.3%, respectively) and no inflammatory changes in the SCoJ, respectively.

### Association between clinical and MRI findings indicative of anterior chest wall inflammation

There was a poor association between clinical and MRI findings indicative of ACW inflammation by the median κ value of all seven individual readers (Table 4). For the SpA group, the agreement between ACW tenderness in at least one of four MASES locations (physician assessment) and BME recorded in at least one of eight ACW joint locations by each of the seven readers showed a median Cohen's κ value of 0.06 (slight agreement; range = 0.00 to 0.20). The association between ACW pain indicated on a manikin form (patient assessment) and BME in at least one of eight ACW joint locations was fair for the SpA (median κ = 0.21, range = 0.06 to 0.29) and the nrSpA group (median κ = 0.33, range = 0.17 to 0.44). There was no association between ACW tenderness and/or ACW pain and either fat infiltration or erosion in at least one of eight ACW joint locations (median κ for fat infiltration = 0.04, range = -0.04 to 0.15; κ = 0.10, range = -0.14 to 0.19) and for erosion (median κ = -0.03, range = -0.11 to 0.01; κ = -0.09, range = -0.18 to 0.17), respectively, in SpA patients.

**Table 4:** Agreement between clinical assessments and MRI-detected lesions of the anterior chest wall indicative of inflammation^a^

**Clinical assessment**^**b**^	**MRI-detected lesion**^**c**^	Patient group	**κ value [range]**^**d**^
ACW tenderness	BME	SpA	0.06 [0.00 to 0.20]
		AS	0.14 [0.06 to 0.20]
		nrSpA	-0.10 [-0.31 to 0.29]
	Erosion	SpA	-0.03 [-0.11 to 0.01]
		AS	-0.03 [-0.11 to 0.03]
		nrSpA	-0.06 [-0.18 to 0.19]
	Fat infiltration	SpA	0.04 [-0.04 to 0.15]
		AS	0.09 [-0.04 to 0.18]
		nrSpA	-0.20 [-0.23 to 0.15]
ACW pain	BME	SpA	0.21 [0.06 to 0.29]
		AS	0.18 [0.03 to 0.28]
		nrSpA	0.33 [0.17 to 0.44]
	Erosion	SpA	-0.09 [-0.18 to 0.17]
		AS	-0.12 [-0.25 to 0.17]
		nrSpA	0.17 [-0.11 to 0.29]
	Fat infiltration	SpA	0.10 [-0.14 to 0.19]
		AS	0.06 [-0.22 to 0.19]
		nrSpA	0.17 [0.07 to 0.44]

^a^ACW = anterior chest wall; AS = ankylosing spondylitis; BME = bone marrow edema; MASES = Maastricht Ankylosing Spondylitis Enthesitis Score [16]; nrSpA = nonradiographic spondyloarthritis; SpA = spondyloarthritis. ^b^ACW tenderness in at least one of four MASES points (physician assessment); ACW pain indicated by patients on a manikin form (patient assessment). ^c^MRI-detected lesion recorded in at least one of eight ACW joint locations by each of the seven readers. ^d^Median Cohen's κ values [range] of all seven individual readers.

## Discussion

In this cross-sectional study, tenderness or spontaneous pain of the ACW was present in 26% of 122 consecutive SpA patients at a tertiary outpatient clinic. Symptomatic patients with long-standing AS and with recent-onset nrSpA were equally affected, indicating that ACW inflammation may start early in the disease course. Compared with these clinical manifestations, involvement by WB MRI was observed more frequently, with 44.3%, 34.4% and 27.0% of patients having BME, erosion and/or fat infiltration, respectively, in at least one location in the ACW. All lesions found by MRI were more common in AS than in nrSpA patients with BME occurring about twice as frequently in the AS group. There was no association between clinical and imaging findings indicative of ACW inflammation.

BME was observed in 9.3%, erosion in 12.0% and fat infiltration in 5.3% of age- and sex-matched healthy controls. This may be due to normal variants or age-related changes. A postmortem microradiographic and histological study of 31 sternal bones showed indentations of the MSJ surfaces in 14 of 20 elderly subjects not affected by rheumatic conditions, as well as herniations of articular cartilage into bone [28]. Both features may mimic erosions of the MSJ on MRI. The poor reproducibility of SClJ erosions may be attributable to often poorly defined cortical margins of normal SClJ seen on MRI studies, due to curved joint surfaces, as well as to the slice thickness used [29]. Another factor mimicking structural SClJ lesions in the usually young SpA population is the multistage ossification of the medial clavicular epiphyseal cartilage between ages 15 and 24, which is used for forensic age diagnostics [30].

In our study, the MSJ was the most frequently affected joint by active and structural lesions detected by MRI and may therefore be regarded as the "index joint" of ACW inflammation. Inflammatory involvement of the MSJ by various imaging modalities has been reported in 39% to 85% of AS patients [4-6]. This wide range may be attributable to different disease duration and severity [31]. In a 10-year retrospective study, radiographic lesions of the MSJ were observed in 51% and of the SClJ in 17% of 76 AS patients [4,5]. The frequencies of BME in 49.5% and of erosion in 36.8% of 95 AS patients in our study are comparable to the detection of active lesions by radionuclide bone scan in 50% and of structural lesions on coronal tomography in 48% of 50 AS patients [6]. The combined imaging modalities in this report also showed a similar distribution of inflammatory lesions among the three ACW joints (SClJ, MSJ and SCoJ involvement in 20%, 32% and 4%, respectively) as in our own study.

We found only moderate association between patient self-report of ACW pain and ACW tenderness upon pressure, with a frequency of ACW involvement of 26% for both evaluations. A combined assessment of patient self-reports and physical examinations containing tenderness and swelling of the ACW reported inflammatory ACW involvement in 58% of 50 AS patients [6].

In our study, we found no agreement between clinical and imaging findings indicative of inflammation, except for a fair association of patient self-report of ACW pain and BME of the ACW. This is in line with a moderate agreement of 36% to 50% between a composite clinical evaluation and a combination of scintigraphic and tomographic findings in 50 AS patients [6]; this study also reported a better association of imaging lesions with spontaneous pain reports than with ACW tenderness. A possible explanation for the discrepancy between clinical and WB MRI findings is subclinical ACW inflammation in SpA patients. This hypothesis is supported by the observation of an increased radioisotope uptake in the SClJ and MSJ in 54% of 50 patients with psoriatic arthritis as opposed to a clinical SClJ involvement in 13% [2]. Subclinical involvement has also been reported at peripheral entheses in SpA [32]. In 34 SpA patients, peripheral enthesitis was detected by clinical examination in 14.4% of the entheses in 62% of the patients compared with 36% of the entheses in 94% of the patients by ultrasonography. A tender point upon clinical examination may not always be indicative of inflamed enthesis and inflammation detected by imaging methods such as ultrasonography and MRI may be clinically silent [33].

Given a paucity of histological data regarding symptomatic enthesitis, imaging may be regarded as the current gold standard for diagnosing this inflammatory condition; however, positive imaging findings may not correlate with clinical enthesitis [34]. Our data were unable to verify the construct validity of the ACW tender points of the MASES [16], which is the only enthesitis score to include this area of the body [17], because no association was observed between clinical and imaging features of inflammation in our study.

The nonspecific characteristic of chest wall pain may also partially account for the poor agreement between clinical and imaging assessment. In a prospective observational cohort study comprising 58 primary care practices in Switzerland, a nonspecific painful chest wall syndrome was found in 1.2% of 24,620 consultations, which recurred in 57% of the patients within 12 months [35]. Some of our SpA patients may have indicated nonspecific chest wall pain unrelated to SpA disease activity, and therefore this may not have been relevant to the inflammation detected by MRI.

A comparison between two different clinical evaluation methods and an imaging assessment has inherent limitations. The patient assessment often shows referred pain distant to sternal inflammation [18-20], whereas the MASES ACW score focuses on two sternal locations. Moreover, the first SCoJ is completely covered by the medial end of the clavicle. The upper location of the MASES ACW score may therefore reflect tenderness of the SClJ or the second SCoJ, which articulates with the MSJ. Although MRI is a sensitive tool for detection of inflammation of the ACW, it may not be surprising that it fails to reveal abnormalities in patients who have nonspecific ACW tenderness on sternal palpation. The assumptions regarding the number of MRI readers and lesions found on MRI studies also affect the comparison with clinical assessment methods.

## Conclusions

Clinical and WB MRI signs of ACW inflammation were found in 26% and in up to 44% of SpA patients, respectively, but there was no association between clinical and imaging findings indicative of inflammation. Consequently, though it is possible that WB MRI may contribute to diagnostic utility, it does not appear to be useful in explaining the cause of a patient's symptoms. This study also raises questions regarding the contribution to the validity of including ACW tender points in an enthesitis score.

## Abbreviations

ACW: anterior chest wall; AS: ankylosing spondylitis; ASAS: Assessment of SpondyloArthritis International Society; BASDAI: Bath Ankylosing Spondylitis Disease Activity Index [23]; BASFI: Bath Ankylosing Spondylitis Functional Index [24]; BASMI: Bath Ankylosing Spondylitis Metrology Index [25]; BME: bone marrow edema; CRP: C-reactive protein, reference range ≤5 mg/L; ESR: erythrocyte sedimentation rate; HLA-B27: human leukocyte antigen B27; MASES: Maastricht Ankylosing Spondylitis Enthesitis Score [16]; MSJ: manubriosternal joint; N/A: not applicable; NRS: numeric rating scale, range 0 to 10; nrSpA: nonradiographic axial spondyloarthritis [13]; SClJ: sternoclavicular joint; SCoJ: sternocostal joint; SpA: spondyloarthritis; STIR: short τ inversion recovery sequence; T1SE: T1-weighted spin-echo sequence; WB MRI: whole-body magnetic resonance imaging.

## Competing interests

The authors declare that they have no competing interests.

## Authors' contributions

AJ, UW, RL, WM, KR and PF drafted the study design. UW and RK acquired the clinical data. AJ, AZ, JH, RL, SD, UW and WM were MRI readers. KR and UW performed the statistical analysis. All authors take responsibility for the interpretation of data. UW drafted the manuscript with contributions from all authors. All authors read and approved the final manuscript.
